# Finite element analysis of the Union Plate in treating elderly acetabular fracture patients

**DOI:** 10.1186/s13018-022-02951-7

**Published:** 2022-01-29

**Authors:** Guixiong Huang, Kaifang Chen, Yulong Wang, Xiaodong Guo

**Affiliations:** Department of Orthopaedics, Wuhan Union Hospital, Tongji Medical College, Huazhong University of Science and Technology, Wuhan, Hubei Province People’s Republic of China

**Keywords:** Acetabular fracture, Static analysis, Transient modal analysis, Osteoporosis

## Abstract

**Background:**

Elderly acetabular fractures are one of the more difficult types of fractures to resolve. For patients at this age, the more common type of fracture is comminuted. How to better fix this type of fracture has always been an issue of concern. This study was performed to observe the mechanical properties of different internal fixation methods used in treating elderly acetabular fracture patients.

**Methods:**

A model of a comminuted acetabular fracture in osteoporosis was established, consisting of an anterior column–posterior hemitransverse fracture with disruption of the quadrilateral surface. Fixation of the acetabular fracture model using a reconstruction plate and Union Plates was simulated. For the different internal fixation methods, static and transient modal analyses were performed under different loads, with an action time of 0.21 s and an analysis time of 0.7 s. The stress of the model was observed in the static analysis, and the displacement of the nodes and the entire model in the U1 direction was observed in the transient modal analysis.

**Results:**

In the static analysis, the stress of the osteoporosis model, the suprapectineal pelvic reconstruction plate model, the infrapectineal quadrilateral surface buttress plate model, and the suprapectineal quadrilateral surface buttress plate model were 42.62 MPa, 37.49 MPa, 44.39 MPa, and 46.15 MPa, respectively. The stress was mainly distributed near the suprapubic branch. The corresponding displacement in the U1 direction was 0.1500 mm, 0.1020 mm, 0.0836 mm, and 0.0990 mm, respectively. In the transient modal analysis, there was a significant difference in displacement between the different models (P < 0.05). When different loads were applied with the same fixation method, there was no significant difference in model displacement (*P* > 0.05).

**Conclusion:**

Static and transient modal analyses show that the infrapectineal quadrilateral surface buttress plate or the suprapectineal quadrilateral surface buttress plate has an advantage in maintaining the stability of fracture fragments when fixing comminuted acetabular fractures in elderly individuals. The infrapectineal quadrilateral surface buttress plate also presents better biomechanical results.

## Introduction

Acetabular fractures in elderly individuals are often accompanied by poor bone conditions caused by osteoporosis [1]. Therefore, comminuted fractures are common in this population and are challenging to treat surgically [2]. Surgery for pelvic fractures is complicated, and open reduction and internal fixation of the pelvis is the current standard for the treatment of acetabular fractures [3]. In addition to the surgical challenge of treating acetabular fractures in the elderly or even trying to restore degenerative changes [4], the condition of such patients is often accompanied by cardiovascular and cerebrovascular diseases, abnormal glucose metabolism, and compensatory liver and kidney function, which will lead to reduced tolerance for surgery [5]. Therefore, there is the potential for many postoperative complications and other factors of uncertainty, often leading to the unsatisfactory results.

Due to the characteristics of acetabular fractures, determining the best surgical approach and method for fixation has always been a challenge. In particular, acetabular fractures in elderly individuals with osteoporosis are commonly comminuted acetabular fractures with dislocation of the femoral head toward the pelvis and compression fractures of the acetabular roof [6]. Achieving strong fixation of comminuted fractures in the quadrilateral surface of ​​the acetabulum is also a challenge in pelvic surgery. In recent years, Lin et al. [7] advocated the use of 3D-printed personalized titanium alloy plates to fix acetabular fractures, which can be used to achieve good fixation of comminuted acetabular fractures. Our team also invented a plate for achieving strong fixation specifically of acetabular fractures, filling this gap, and used sawbones to perform related mechanical tests, which showed good results [8].

The mechanics of internal fixation in the human body are complicated. The external forces placed on internal fixation instrumentation in the human body may be different when the body is at rest and when it is moving. To study the force at rest and at a certain moment during activity, as well as the change in the response over time after the external force ends, the finite element method was used to study an acetabular plate that we designed, i.e. the Union Plate, in the treatment of comminuted acetabular fractures in elderly patients with osteoporosis and to compare it with traditional internal fixation methods.

## Method

**Fig. 1:**
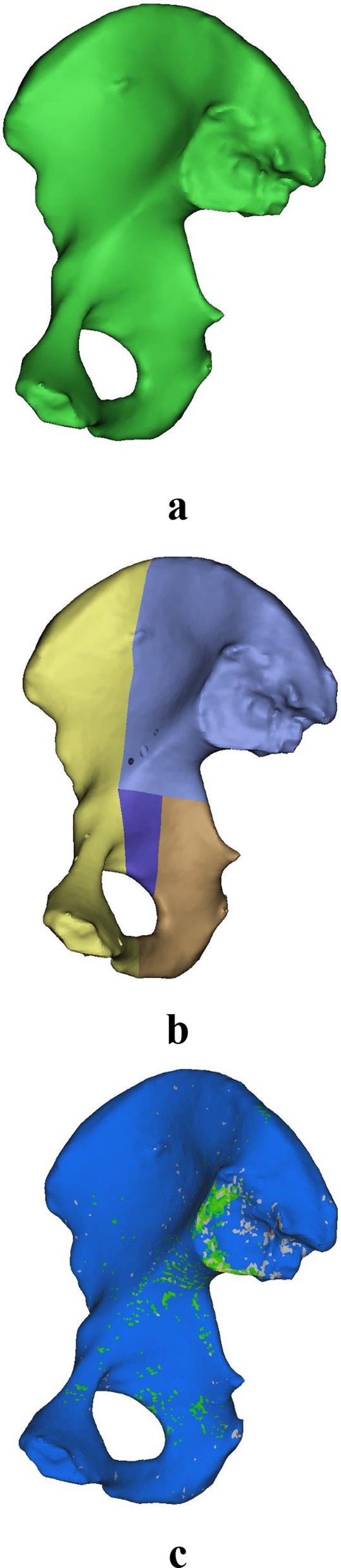
**a** A right iliac osteoporosis model, **b** a right iliac osteoporosis acetabular comminuted fracture model, and **c** a right iliac osteoporosis model with assigned materials

**Fig. 2:**
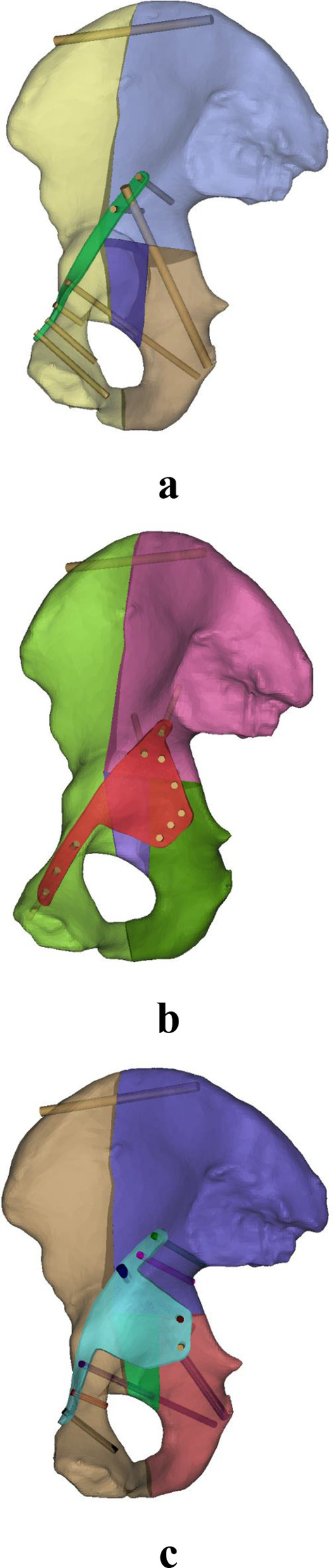
**a** A suprapectineal pelvic reconstruction plate, **b** a infrapectineal quadrilateral surface buttress plate, and **c** a suprapectineal quadrilateral surface buttress plate

The computed tomography (CT) data of an elderly female volunteer (72 years old) who was diagnosed with osteoporosis and had no other diseases that might cause bone loss were imported into Mimics 20.0 (Materialise, Leuven, Belgium) for 3D reconstruction. This research was ethically approved by the Tongji Medical College of Huazhong University of Science and Technology (No. S1060). The goal of this study was to perform a mechanical analysis of acetabular fractures in elderly patients after surgery; the right ilium of the pelvis was reconstructed, and the influence of the surrounding soft tissues was ignored. Subsequently, a comminuted acetabular fracture model, consisting of an anterior column–posterior hemitransverse fracture with disruption of the quadrilateral surface, was established using the reconstructed osteoporotic right ilium. In 3-matic 12.0, models of a suprapectineal pelvic reconstruction plate (SPRP), an infrapectineal quadrilateral surface buttress plate (IQSBP, simplified model of the Union Plate), and a suprapectineal quadrilateral surface buttress plate (SQSBP, simplified model of the Union Plate) were established. After the plates were placed and the corresponding screws inserted, the models were imported into HyperMesh 13.0 (Altair Company, Troy, MI, USA) in *stl* format for meshing. The final SPRP model had 6017 elements and 1954 nodes; IQSBP, 18,637 elements and 5437 nodes; and SQSBP, 16,417 elements and 4888 nodes. The fracture model, without the plates, was imported into Mimics 20.0 for material assignment based on the CT values. This study assumed that the bone and titanium plate were isotropic materials. The material assignment formula was built in Mimics 20.0, and Young's modulus (*E*) was calculated based on the CT values, as follows: 1. grey value ≤ 699 HU: *ρ* = 0.35, *E* = 4000, Poisson's ratio (*v*) = 0.3; and 2. grey value > 699HU: *E* = − 22,000 + 24,000*ρ, *v* = 0.3. The materialized models were then imported into Abaqus 21.0 (Dassault; France) for mechanical analysis, as shown in Figs. 1 and 2. The plates and screws were manually assigned materials in Abaqus 21.0, and the material properties were assigned according to a study by Cun et al. [9].

The contact between fractured bone pieces was set to face-to-surface contact; the tangent behaviour was set to hard contact; the contact attribute was set to penalty friction; and the coefficient was 0.3. The contact between bone and plate was set as face-to-surface contact; the tangent behaviour was set to hard contact; the contact attribute was set to penalty friction; and the coefficient was 0.45 [10]. Because in the actual operation, there is very close contact between the plate and screws, they can be regarded as a whole. The contact relationship between the plate and screws was set as a binding relationship. Six degrees of freedom of the sacroiliac joint and the pubic symphysis were fixed. The research procedure was divided into two parts, static analysis and transient modal analysis.

### Static analysis

The loading method applied a concentrated force of 1000 N to each model at an angle of 45° from the horizontal direction (along the X-axis), acting on the acetabular fossa to simulate the force of the acetabulum. The main measure was the maximum displacement of nodes in the U1 direction (along the X-axis) after the force was applied to the osteoporosis model (OM) with the SPRP, IQSBP, or SQSBP fixation. Additionally, the maximum stress and stress distribution of the OM and all plates were observed and recorded.

### Transient modal analysis

Transient modal analysis [11] is a kind of dynamic analysis, in which an external load acts on an object for a short time or is in a dynamic manner. This part of the study was performed to observe the shielding effect of the internal fixation methods on the acetabulum under the dynamic action of different external loads under the acetabular fossa by exploring the displacement of nodes of interest of the model in the direction of the force. The external load was gradually increased; the action time was 0.21 s (the force increased from 0 to the peak in the first 0.01 s, and the load peak time was 0.2 s), and the analysis time was 0.7 s. In this study, the analysis time was set to be more than three times the load action time to facilitate the observation of the response of the model after the removal of the external load. In dynamics, dynamic simulation includes the inertial force in the dynamic equation:

1 $$M\ddot{u} - P + I = 0$$

where *M* is the mass of the structure, $\ddot{u}$ is the acceleration of the structure, *I* is the internal force of the structure, and *P* is the applied load. In this study, the external load was applied for a short time. If a load is consistent with or close to the natural frequency of an object, it will have a destructive effect on the object [12]. Therefore, the minimum natural frequency value of the model needs to be obtained after modal analysis combined with observation, and the loading frequency needs to be far away from the minimum natural frequency. The formula for calculating the natural frequency of an object is as follows:

2 $$\omega = \sqrt{\frac{k}{m}}$$

where *m* is the mass of the object and *k* is the stiffness of the object. According to formula (1), if an external force is applied to an object, the object will vibrate at frequency *ω*. If the frequency of the object subjected to the external force is consistent with the natural frequency of the object, the amplitude of the displacement will become more intense. This phenomenon is called resonance [13]. For determination of the natural frequency of an object, let *P* (external force) = 0 in formula (1); then, the motion equation (formula (1)) becomes the following:

4 $$M\ddot{u} + I = 0$$

Assuming that the motion system has no damping, *I* = *Ku*, where *K* is the stiffness and *u* is the displacement. Then, formula (4) becomes the following:

5 $$M\ddot{u} + Ku = 0$$

The solution of this equation has the form:

6 $$u = \phi {\text{e}}^{{{\text{i}}\omega t}}$$

where *φ* is the eigenvector and the eigenvalue (*λ*) can be obtained by bringing (6) into (5):

7 $$K\phi = \lambda M\phi$$

where *λ* = *ω*^*2*^. The dynamic system has n eigenvalues, where n is the number of degrees of freedom in the finite element model (such as all models in this study). *λ *_*j*_ is the *j* eigenvalue, its square root *ω*_*j*_ is the natural frequency of the structure in the *j* order mode, and *φ*_*j*_ is the corresponding *j*th order eigenvector, which is the mode, that is, the mode shape, because it is the structure with the *j*-order deformed mode shape of the vibration. In this study, we extracted the modal shape and natural frequency of the first 30 orders of the model. These two factors were used to qualitatively and linearly analyse the dynamic response under loading; additionally, the mode superposition technique [14] can be used to calculate the deformation of the model.

After the static analysis, the original model was copied in Abaqus 2021, and the frequency and modal dynamics analysis steps were recreated in the analysis step module. In the frequency analysis step, the Lanczos solver was used to extract the eigenvalues of the first 30 orders. In the modal dynamic analysis step, the analysis time was set to 0.7 s. After the experiment, the time increment was set to 0.001, and system damping [15] and direct modal damping were used in this study.

In the transient modal analysis, concentrated forces of 600 N, 800 N, 1000 N, 1200 N, 1400 N, and 1600 N at an angle of 45° from the horizontal direction were applied to the acetabular fossa of all models [16]. The action time of the concentrated force was 0.21 s, and the maximum displacement values of the OM and SPRP, IQSBP, and SQSBP models in the U1 direction were obtained.

Statistical analysis was performed using multifactor analysis of variance. The standard deviation and variance were used to determine whether there were significant differences between various models. *P* < 0.05 was considered to indicate a significant differences. PyCharm (Community Edition 2021.1.1) was used for statistical data processing.

## Results

The maximum displacement in the U1 direction in the static analysis and the transient modal analysis of each model is shown in Table 1.

**Table 1:** Maximum displacement of model U1 direction (mm)

	Statics analysis	Transient modal analysis					
	F = 1000 N	F = 600 N	F = 800 N	F = 10000 N	F = 1200 N	F = 1400 N	F = 1600 N
OM	0.1500	0.4370	0.5830	0.7290	0.8750	1.0200	1.0200
SPRP	0.1020	0.0300	0.0410	0.0510	0.0610	0.0710	0.0810
IQSBP	0.0836	0.0137	0.0183	0.0229	0.0275	0.032	0.0366
SQSBP	0.09901	0.0227	0.0303	0.0378	0.0454	0.053	0.0605

**Fig. 3:**
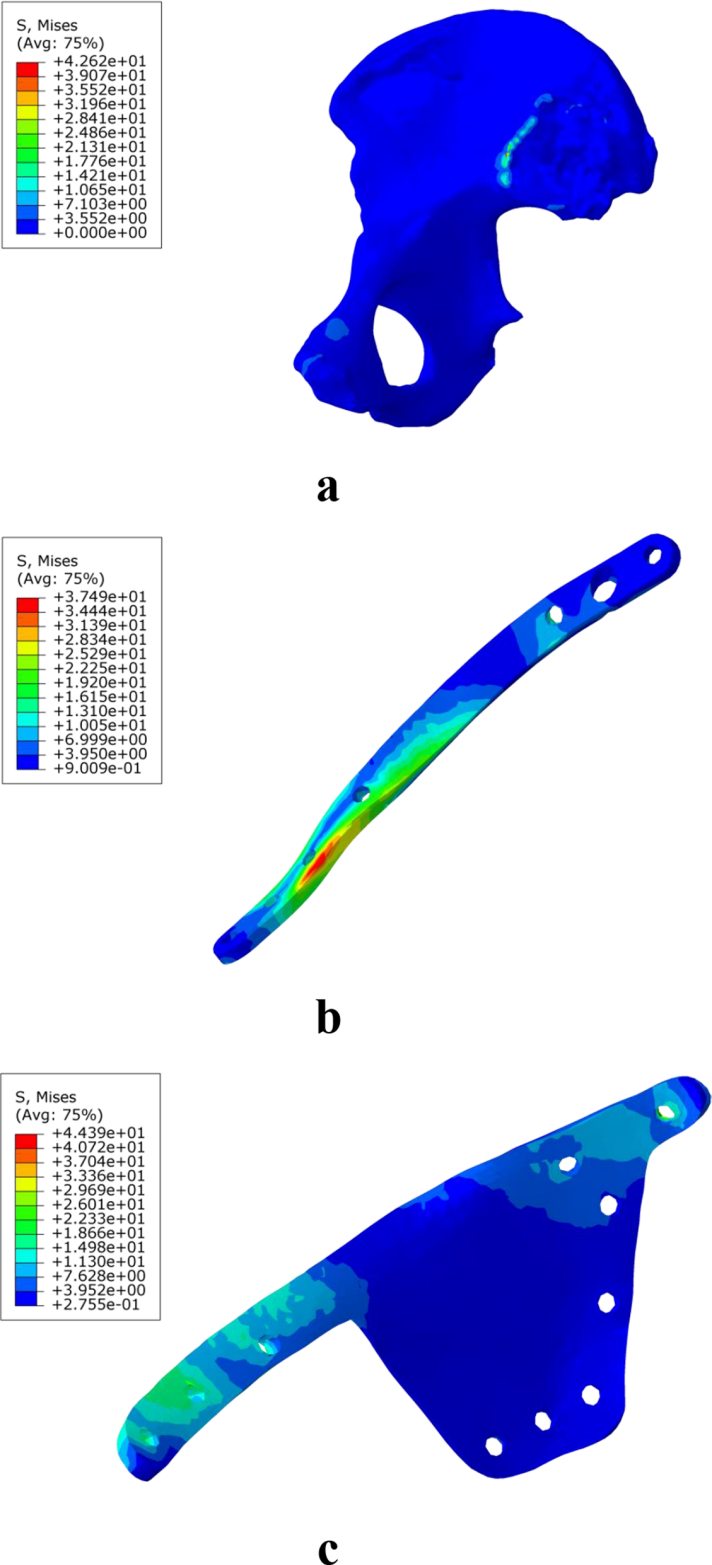

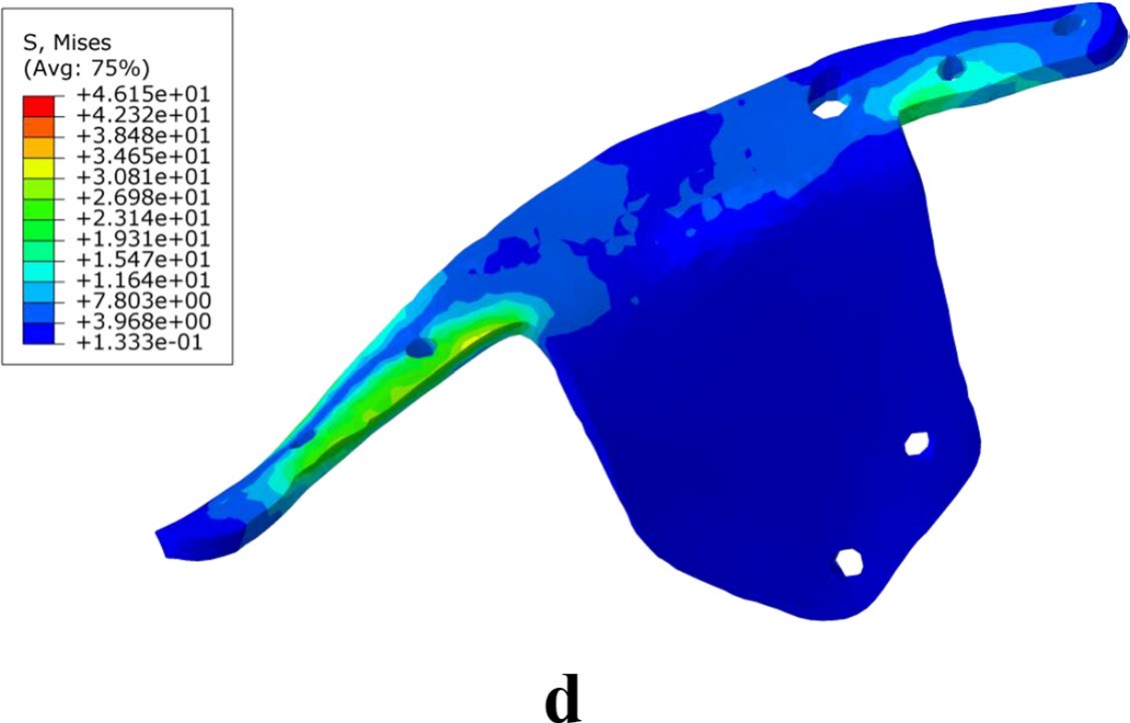
Stress distribution diagram of the model during static analysis (**a** is OM, an osteoporosis model, **b** is SPRP, a suprapectineal pelvic reconstruction plate, **c** is IQSBP, an infrapectineal quadrilateral surface buttress plate, and **d** is SQSBP, a suprapectineal quadrilateral surface buttress plate)

In the static analysis, the stress of the OM and SPRP, IQSBP, and SQSBP models was 42.62 MPa, 37.49 MPa, 44.39 MPa, and 46.15 MPa, respectively. The stress was distributed in the ipsilateral sacroiliac joint of the pelvis in the OM, in close proximity to the suprapubic branch above the obturator in the SPRP model, at the suprapubic branch in the IQSBP model, and at the internal fixation of the suprapubic branch at the junction of the acetabular quadrilateral surfaces in the SQSBP model (Fig. 3).

**Fig. 4:**
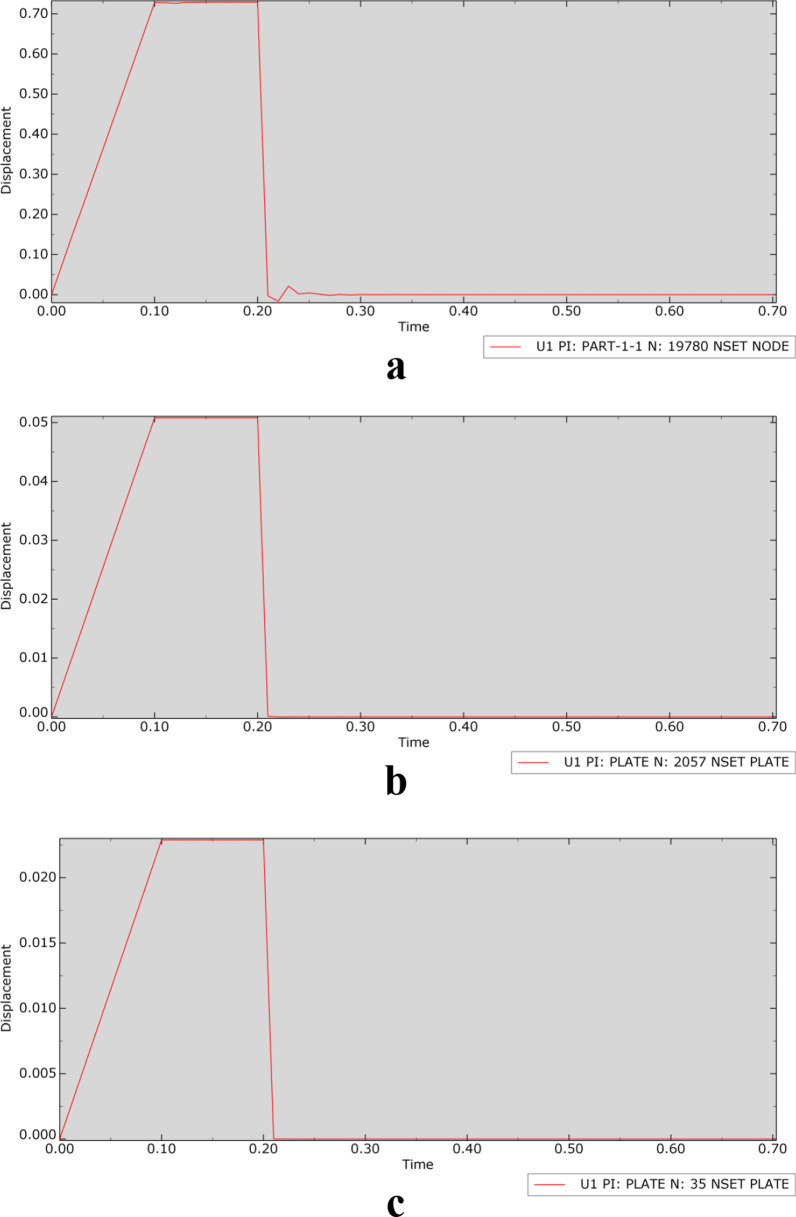

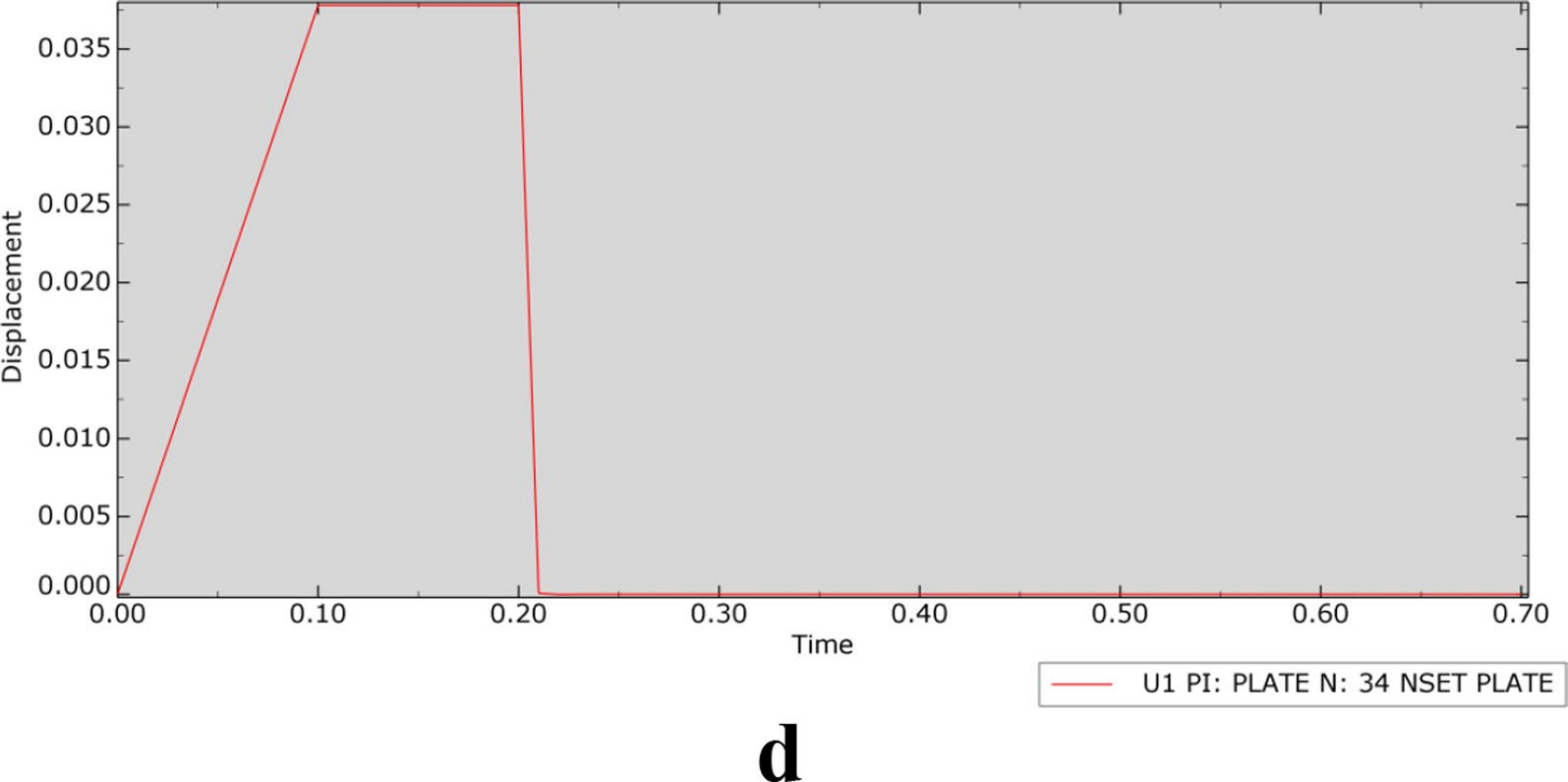
Displacement–time curve (**a** is OM, an osteoporosis model, **b** is SPRP, a suprapectineal pelvic reconstruction plate, **c** is IQSBP, an infrapectineal quadrilateral surface buttress plate, and **d** is SQSBP, a suprapectineal quadrilateral surface buttress plate)

In the transient modal analysis of each model, the relationship between displacement and time was determined, as shown in Fig. 4 (taking an applied load of 1000 N as an example).

Multifactor analysis of variance showed a significant difference in the displacement in the U1 direction among different models under different forces during the static and transient modal analyses (*P* = 3.7 × 10^–5^, *P* < 0.05). In the static and transient modal analyses of loads applied to the same model, statistical analysis yielded a *P* value of 0.1145 (*P* > 0.05), indicating no significant difference in the displacement in the U1 direction under different loads applied to the same model. Comparison of the displacement in the U1 direction under different loads showed a significant difference between the OM and SPRP, IQSBP, and SQSBP models. The SPRP, IQSBP, and SQSBP models were compared in pairs, and the results indicated no significant differences between them. The standard deviation and variance of the models were as follows: OM, 0.3216 and 0.1034; SPRP, 0.0246 and 0.0006; IQSBP, 0.0234 and 0.0005; and SQSBP, 0.0252 and 0.0006. The standard deviation and variance of the IQSBP model were the smallest.

## Discussion

The results of this experiment show relatively large displacement values in the OM and SPRP model in both the static and transient modal analyses. At the same time, the displacement values in the IQSBP and SQSBP models are relatively small, indicating that when the acetabular area is subjected to external force, the IQSBP and SQSBP play a shielding role. On statistical analysis, the OM without internal fixation was significantly different from the other fracture models with internal fixation. The results also suggest that better performance in terms of stabilization was achieved with IQSBP fixation. In addition, an interesting phenomenon was observed: when the external force reached 1400 N, the nodes of the OM did not show a further increase in the maximum displacement, while those of the SPRP, IQSBP, and SQSBP models still showed signs of continued increases in displacement. This phenomenon may indicate that when an object is subjected to an external force, as the load increases, the displacement increases to a certain extent. However, when the force increases to a certain threshold, the displacement will no longer continue to increase. If the load continues to increase beyond this point, the load will have a destructive effect on the material, which is the ultimate stress of the material [17]. Because of the shielding effect of IQSBP and SQSBP fixation on the acetabulum, the displacement did not fluctuate sharply as the load increased, approaching the limit stress. This phenomenon further verified the shielding effect of IQSBP and SQSBP fixation on the acetabular area.

According to the displacement–time curve, when the force disappeared at 0.21 s, there was still a relatively small vibration in the OM, while there was no vibration in the other fracture models with internal fixation after the load disappeared. This phenomenon further verified the stabilizing effect of internal fixation on the fracture.

In this study, we noticed that the stress of the OM model was distributed on the sacroiliac joint. After analysing the material distribution of the model, the elastic modulus of bone in this area is relatively high, so the stress was concentrated here. This is one possible reason why crescent fractures [18] can occur during treatment. The stress of the SPRP and IQSBP models was mainly distributed on the screws close to the pubic symphysis, which may be one reason for complications of internal fixation of the pubic symphysis, such as screw loosening, pull-out, or breakage, in clinical practice [19, 20]. The stress of the SQSBP model was also at the upper pubic ramus, at the junction where the internal fixation instrumentation extended to the quadrilateral surface of the acetabulum. These results support the preliminary conclusion that stress concentration is more likely to occur at the upper pubic ramus, which may be due to drastic changes in the anatomical geometry [21]. Pelvic fractures are often accompanied by fractures of the suprapubic ramus, which also supports this conclusion to some extent. Therefore, internal fixation instrumentation at this location is prone to stress concentration, resulting in screw breakage and loosening.

In this study, statistical analysis of the different analysis methods showed in difference between the OM and the other models in displacement, but no significant difference among the internal fixation models. There was also no significant difference among the different loads applied to the same model. Analysis of the standard deviation and variance suggested that IQSBP fixation has an advantage in terms of mechanics, while no significant difference among SPRP, IQSBP, and SQSBP fixation was found on multivariate analysis of variance. However, when evaluating a model of internal fixation, the clinical significance of various factors usually needs to also be considered. The displacement–time curve indicates that SPRP fixation is not suitable, suggesting poor shielding of the acetabular quadrilateral surface. This performance is unacceptable in elderly patients with osteoporosis and comminuted fractures of the acetabulum. The shielding effect on the acetabular quadrilateral surface of IQSBP fixation is better than that of all other modelled internal fixation methods, but our research showed stress concentration at the screw near the pubic symphysis, reaching 64.42 MPa, which is a critical disadvantage. This kind of situation needs to be considered clinically because such stress concentration may lead to postoperative screw breakage. This is especially true in young patients who are at greater risk than elderly patients because of their higher activity levels. The shielding effect on the quadrilateral surface of IQSBP fixation was between that of SPRP and IQSBP fixation, although the maximum stress was distributed near the suprapubic branch, at the junction of the suprapubic branch and the acetabular quadrilateral surface. Furthermore, the maximum displacement of this model was the smallest among all the other internal fixation models, which serves as a reminder to optimize the product to disperse the stress. In summary, we recommend IQSBP fixation as the first choice in these cases.

Elderly acetabular fracture patients have their own characteristics. First, due to vascular sclerosis, elderly individuals are prone to blood vessel rupture due to the transition of traction during the operation, which increases the potential risk of bleeding [22]. Second, elderly individuals have more systemic diseases, limiting the duration of the operation. Finally, most acetabular fractures in elderly individuals are comminuted fractures [23], which cannot be fixed using ordinary plates. Acetabular fractures in elderly individuals are not the same as those in other individuals. If the "seagull sign" appears, the main goal is often to repair the acetabular roof, and hip replacement may also be required in the future [24].

Our invention (the Union Plate) meets all the requirements for the ideal internal fixation for acetabular fractures in elderly individuals. In this study, the application of this SQSBP, with special screw holes designed for the subacetabular screw and posterior column plate, can reduce the time required for the operation, which is very beneficial for elderly individuals. In the case of a comminuted fracture of the quadrilateral surface, the Union Plate can be applied to fix the fracture through the subacetabular and posterior column screws, forming a fixed frame structure. In addition, despite the fragments of the quadrilateral surface, the SQSBP plays a shielding role, based on the "frame-buttress theory" we have proposed. Moreover, the plate is anatomically designed based on big data, conforms to the bone morphology of most people in China, and fits well with the bone surface [25].

Of course, IQSBP and SQSBP fixation have their shortcomings. Because the plates are relatively large, there are certain difficulties in intraoperative placement, necessitating a highly skilled surgeon. Furthermore, when using these plates, it is necessary to fully expose the quadrilateral surface during the surgical approach. Therefore, we recommend the use of a high inguinal approach or pararectus abdominis approach [26]. Finally, these plates easily damage the surrounding blood vessels and nerves. Thus, we recommend performing an enema and reducing the intra-abdominal pressure before surgery. These plates are not suitable for fractures of the posterior wall of the acetabulum.

### Limitation

One limitation of this study is that only a part of the pelvis was considered, without the effects of ligaments or muscles; additionally, the bone was set as an isotropic material, while bone is in fact an anisotropic material [27]. Furthermore, when the load was applied, the yield stress of the bone was not considered [28], and further research is needed to determine whether middle-aged and elderly people with osteoporosis can tolerate a force of 1600 N.

## Conclusion

The displacement of the OM increased with increasing external load, but after a certain point, even though the external load continued to increase, the displacement did not increase. The suprapubic branch of the pelvis is an area where mechanical stress can concentrate easily. Considering various factors, such as the presence of osteoporosis, characteristics of comminuted fractures, limitations of traditional plates, and observed biomechanical results, IQSBP fixation may have an advantage in maintaining the stability of fracture fragments when used to treat comminuted acetabular fractures in elderly individuals.

## Data Availability

Available upon request from the corresponding author.

## Abbreviations

CT: Computed tomography
SPRP: Suprapectineal pelvic reconstruction plate
IQSBP: Infrapectineal quadrilateral surface buttress plate
SQSBP: Suprapectineal quadrilateral surface buttress plate
OM: Osteoporosis model
